# CD4 T-Cell Enumeration in a Field Setting: Evaluation of CyFlow Counter Using the CD4 Easy Count Kit-Dry and Pima CD4 Systems

**DOI:** 10.1371/journal.pone.0075484

**Published:** 2013-09-16

**Authors:** Djibril Wade, Papa Alassane Diaw, Géraldine Daneau, Makhtar Camara, Tandakha Ndiaye Dieye, Souleymane Mboup, Luc Kestens

**Affiliations:** 1 Immunology Unit, Laboratory of Bacteriology Virology, Le Dantec University Teaching Hospital, Dakar, Senegal; 2 Immunology Laboratory, Department of Biomedical Sciences, Institute of Tropical Medicine, Antwerp, Belgium; 3 Department of Biomedical Sciences, University of Antwerp, Antwerp, Belgium; University of New South Wales, Australia

## Abstract

**Background:**

Flow Cytometry (FCM) is still considered to be the method of choice for accurate CD4 enumeration. However, the use of FCM in developing countries is problematic due to their cost and complexity. Lower-cost technologies have been introduced. We evaluated CyFlow Counter together with its lyophilized reagents, and Pima CD4 in high-temperature area, using FACSCount as reference.

**Materials and Methods:**

Whole blood samples were consecutively collected by venipuncture from 111 HIV+ patients and 17 HIV-negative donors. CD4 T-cell enumeration was performed on CyFlow Counter, Pima CD4 and FACSCount.

**Results:**

CyFlow Counter and Pima CD4 systems showed good correlation with FACSCount (slope of 0.82 and 0.90, and concordance ρ_c_ of 0.94 and 0.98, respectively). CyFlow Counter showed absolute or relative biases (LOA) of −63 cells/mm^3^ (−245 to 120) or −9.8% (−38.1 to 18.4) respectively, and Pima CD4 showed biases (LOA) of −30 cells/mm^3^ (−160 to 101) or −3.5% (−41.0 to 33.9%). CyFlow Counter and Pima CD4 showed respectively 106.7% and 105.9% of similarity with FACSCount. According to WHO-2010 ART initiation threshold of 350 cells/mm^3^, CyFlow Counter and Pima CD4 showed respectively sensibility of 100% and 97%, and specificity of 91% and 93%. CyFlow Counter and Pima CD4 were strongly correlated (slope of 1.09 and ρ_c_ of 0.95). These alternative systems showed good agreement with bias of 33 cells/mm^3^ (−132 to 203) or 6.3% (−31.2 to 43.8), and similarity of 104.3%.

**Conclusion:**

CyFlow Counter using CD4 easy count kit-dry and Pima CD4 systems can accurately provide CD4 T-cell counts with acceptable agreement to those of FACSCount.

## Introduction

In the last decade, expanding access to treatment has contributed to a significant decline in deaths among people living with HIV/AIDS. However, only 37% of individuals in need currently receive antiretroviral therapy (ART) [1]. In most resource-limited countries, CD4 T-cell enumeration is the most critical laboratory assay used to initiate the chemoprophylaxis against opportunistic infections. It is also used to start the ART and to evaluate the therapeutic effectiveness. Flow Cytometry (FCM) is considered as the reference method for CD4 T-lymphocyte enumeration [2]–[5]. However, the flow cytometers and their maintenance remain expensive. The use of FCM requires well-trained staff and a cold chain to ship and store reagents. Thus, the flow cytometers are not as widely spread for routine CD4 measurements in resource-limited as in developed countries. To ensure continuation of scaling-up of ART in resource-limited areas, simpler and cheaper methods for CD4 T-cell enumeration are used instead [6], [7].

The FACSCount (Becton Dickinson, San Jose, CA) is the oldest dedicated flow cytometer that has been extensively validated in resource-limited settings [8], [9]. It is still widely used, but remains expensive.

Over the past decade, more affordable CD4 T-cell counters have been developed, mostly based on Flow Cytometry: CyFlow SL Blue®, CyFlow Green®, CyFlow Counter® (Partec GmbH, Münster, Germany), Guava EasyCD4 (Guava Technologies, Hayward, CA), Apogee Auto40 (Apogee Flow Systems, Hemel Hempstead, UK), Pointcare Now® (Pointcare Technologies, Marlborough, MA) except the recent Point-of-Care (POC) Pima CD4 system (Alere, Jena, Germany) which is based on digital image analysis. The introduction of these POC CD4 technologies will reduce the proportion of patients lost to follow-up, and facilitate early ART initiation by healthcare workers as it will reduce the exclusive dependency on clinical staging [10]–[14]. Several studies have evaluated these alternative CD4 T-cell assays. Most of them were conducted under optimal conditions, different from real field conditions of laboratories in resource-limited areas where alternative CD4 systems would be used [12], [15]–[21].

Recently, Partec developed dry lyophilized CD4 reagents, the “CD4 easy count kit-dry” for use on the CyFlow Counter. The CyFlow Counter is a fully equipped ultra-compact FCM with a solid green laser. The CyFlow Counter (CY-S-3021) can provide absolute CD4, CD8 and CD3 T-cell counts. The most recent model of CyFlow Counter (CY-S-3022) (http://www.partec.com/instrumentation/products.html?&tx_cyclosproductfinder_cyc_pf_display[product] = 1017&tx_cyclosproductfinder_cyc_pf_display[action] = show&tx_cyclosproductfinder_cyc_pf_display[controller] = Product) is a portable and compact flow cytometer dedicated for routine absolute CD4 T-cell counting (by Primary gating) and measurements of CD4 percentages (by Panleucogating). Alere developed a portable battery-powered POC system, the Pima CD4 (http://pimatest.com/en/pima-platform/pima-analyser.html), which counts absolute CD4 T-lymphocytes in either finger-prick or venous blood sample within 20 minutes using dedicated thermostable cartridges. The Pima CD4 is equipped with miniaturized multi-colour fluorescence imaging optics. Both instruments do not require the presence of a cold chain and stable regular power supply and are therefore suitable for use at resource-limited places. However, their accuracy and precision have not yet been evaluated under field conditions.

In this study, we aimed to evaluate the current model of CyFlow Counter (CY-S-3022) together with its lyophilized reagents (CD4 easy count kit-dry, for Primary gating) and the Pima CD4 system in a rural district laboratory in Ziguinchor, Senegal using FACSCount as reference for absolute CD4 counts. Furthermore, comparing 2 alternative POC systems based on different technologies (flow cytometry versus digital image analysis) between them and with a third reference technology helps to interpret discordant results. While there is no true gold standard for CD4 measurement, an error can indeed be caused by the reference itself, and not always by the evaluated instrument.

## Materials and Methods

### Ethics statement

The Ethical Committees waived the need of informed consent as we used the excess of routine blood samples from anonymous participants. Only age, sex, HIV-profile and time of blood collection were collected from blood samples included in this study. HIV-positive samples were collected from patients attending their normal CD4 T-cell monitoring. HIV-negative samples were collected from blood donors at the blood bank. This study was approved by the Senegalese National Ethical Committee of the Ministry of Health (Dakar, Senegal) and by the Institutional Review Board of the Institute of Tropical Medicine (Antwerp, Belgium).

### Blood samples

Study participants were consecutively recruited among the patient population attending the Regional Hospital and 2 Healthcare Centres (Silence HC and Bignona HC) in September 2011 in Ziguinchor, Senegal. Whole blood samples were collected by venipuncture into K_3_ EDTA-containing tubes from 111 anonymous HIV-infected patients from the 3 sites for their routine immunological follow-up, and 17 anonymous HIV-negative donors from the blood bank at the Regional Hospital. Due to anonymity, ART status was not known.

### CD4 T-cell enumeration

CD4 counts were determined in whole blood samples within 6 hours after venipuncture, on three different instruments according to the manufacturer's instructions: on FACSCount and Pima CD4 at the Regional Hospital, and on CyFlow Counter at the Silence HC. Samples were put in a safe container, and transportation between both laboratories lasted 15 to 20 minutes. CD4 T-cell counting was performed by different operators (blind reading).

The FACSCount is a fully equipped single platform (SP) FCM equipped with a green laser. It is combined with built-in software and two-colour monoclonal antibodies (mAb) reagents in a twin-tube containing calibrated beads, with additional control beads. Briefly, 50 mm^3^ of EDTA anti-coagulated whole blood was added to the FACSCount reagent tubes containing anti-CD3-PE, and anti-CD4-PE-Cy5 or anti-CD8-PE-Cy5. The tubes were capped, vortexed and incubated in the dark at room temperature for 60 minutes. After incubation, 50 mm^3^ of fixative solution was added to reagent tubes, and samples were run on the FACSCount instrument. That FACSCount is certified by the Quality Assessment and Standardization of Immunological Measures Relevant to HIV/AIDS (QASI) with the FACSCount reagents that only provide absolute CD4 counts.

The model used in our study, the CY-S-3022 was used with the CD4 easy count kit-dry based on ready-prepared test tubes containing “dry” lyophilized CD4-PE mAb for volumetric absolute measurements. The CyFlow Counter was installed at the Silence HC's laboratory. The procedure consists of adding 20 mm^3^ of whole blood into reagent tube followed by gently mixing and incubation at room temperature in the dark for 15 minutes. The buffer solution was added, and samples were run on the CyFlow Counter.

The Pima CD4 was placed at the Regional Hospital into a room without air-conditioner, where outside ambient temperature varied between 33 to 37°C. According to manufacturer's recommendations, 25 mm^3^ of blood sample was pipetted into a disposable anticoagulant-coated cartridge preloaded with anti-human CD3-dye1 and CD4-dye2 mAb. Once the window was filled, we removed the collector. The cartridge was capped and immediately inserted into Pima analyser to run test.

### Precision assessment

Intra-assay precision was assessed by repeating 10 times the entire CD4 staining procedure on 3 blood samples with clinically relevant CD4 counts (<200; 300–500; >500 cells/mm^3^). Besides, specific control beads were daily run on the CyFlow Counter and Pima CD4. These values (14 each) were used to calculate the instrumental precision.

### Statistical analysis

Data analyses were done using MedCalc 10.0.2.0 (MedCalc Software, Mariakerke, Belgium). Precision expressed as the coefficient of variation (CV) was determined by dividing the standard deviation (SD) of the 10 measurements by the mean (%CV = SD×100/mean). Measurement of linear regression was determined using Passing-Bablok regression analysis, which, in common with all non-parametric methods, is less sensitive to outliers [22]. The concordance correlation coefficient (ρ_c_ = ρ×C_b_) evaluates the degree to which pairs of observations fall on the 45° line through the origin [23]. In this equation, the Pearson correlation coefficient ρ could be considered as a surrogate parameter of “precision” and bias correction factor C_b_ as a surrogate marker of “accuracy”. Bland Altman and Pollock analyses were used to analyse the agreement between methods. Relative bias, which is more useful to compare absolute counts between two methods, was plotted for each sample against the average. Mean bias and limits of agreement (LOA) as mean bias ±1.96×SD were calculated [24], [25]. The percentage similarity was calculated for each sample as% Similarity  =  Average of Methods A and B×100/Method A (with A =  reference method, B =  new method). For each group, the mean similarity and the relative SD were determined [26].

Comparisons were done between alternative methods (CyFlow Counter or Pima CD4) and FACSCount, and between both alternative methods. Both alternative systems were simultaneously compared against the reference by plotting bias of CyFlow Counter and Pima CD4 regarding to FACSCount against FACSCount CD4 counts in a same graph.

Overall results and those of HIV-infected patients (HIV^+^) alone were compared. Data from the 3 clinically relevant CD4 ranges (Low CD4 as <200 cells/mm^3^, Medium CD4 as between 200 and 500 cells/mm^3^, and High CD4 as >500 cells/mm^3^) were separately compared.

For clinical significance of the measurement differences on treatment decision, inter-rater agreement was used to calculate the kappa coefficient on the HIV^+^ patients [27]. Sensibility and specificity values were calculated at the ART eligibility CD4 thresholds of 200, 350 and 500 cells/mm^3^ for both methods. FACSCount CD4 results were set as the reference to determine eligible patients on ART.

## Results

### Study population

A total of 128 whole blood samples were included in this study: 99 HIV-1, 9 HIV-2, 3 HIV-1+2-infected patients, and 17 HIV-negative donors. The median age was 38 years (range: 7–70) and the female sex percentage was 78%. The median (min – max) absolute CD4 counts provided by FACSCount, CyFlow Counter and Pima CD4 were respectively 404 cells/mm^3^ (1–1798), 346 cells/mm^3^ (4–1542) and 369 cells/mm^3^ (7–1753).

### Precision analysis and instrumental repeatability

FACSCount and CyFlow Counter systems showed a mean intra-assay precision, expressed as CV, lower than 6%, while Pima CD4 showed CV greater than 10% (Table 1).

**Table 1:** Inter-assay precision of FACSCount, CyFlow Counter and Pima CD4.

	N	Low CD4	Medium CD4	High CD4
FACSCount: Mean; V; (SD)	10	143 cells/mm^3^; 5.25%; (8 cells/mm^3^)	360 cells/mm^3^; 5.95%	766 cells/mm^3^; 4.02%
CyFlow Counter: Mean; CV; (SD)	10	148 cells/mm^3^; 9.83%; (14 cells/mm^3^)	350 cells/mm^3^; 4.07%	639 cells/mm^3^; 4.03%
Pima CD4: Mean; CV; (SD)	10	163 cells/mm^3^; 17.61%; (29 cells/mm^3^)	381 cells/mm^3^; 10.52%	682 cells/mm^3^; 10.76%

N means Number of replicates; CV means Coefficient of Variation; SD means absolute standard deviation (presented for low CD4 only); Low means CD4 lower than 200 cells/mm3; Medium means CD4 between 300 and 500 cells/mm3; and High means CD4 greater than 500 cells/mm3.

Additionally, specific control beads were daily analysed on CyFlow Counter (*Count Check Beads green*) and Pima CD4 (*Low* and *Normal beads controls*). Controls were successfully passed. Instrumental precision (CV) determined with control beads was lower than 3% for both CyFlow Counter and Pima CD4.

### Comparison between alternative methods and the reference

**Figure 1:**
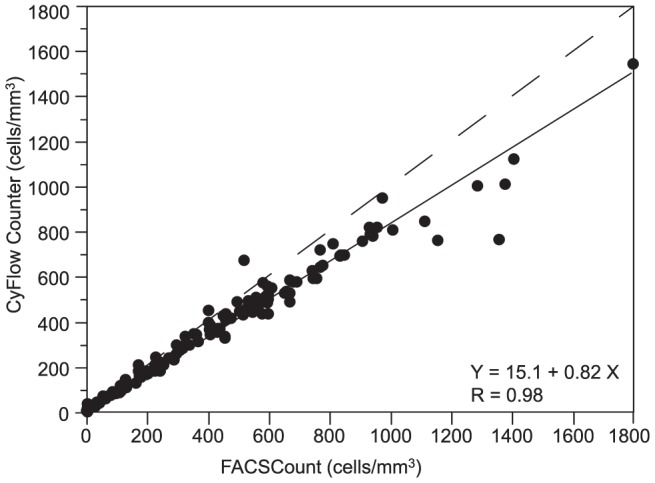
Passing-Bablok regression between CyFlow Counter and FACSCount (N = 128). The x-axis represents CD4 counts provided by the FACSCount reference and the y-axis represents the CD4 counts provided by the CyFlow Counter. The solid line represents the regression line, and the dashed line represents the line y = x.

**Figure 2:**
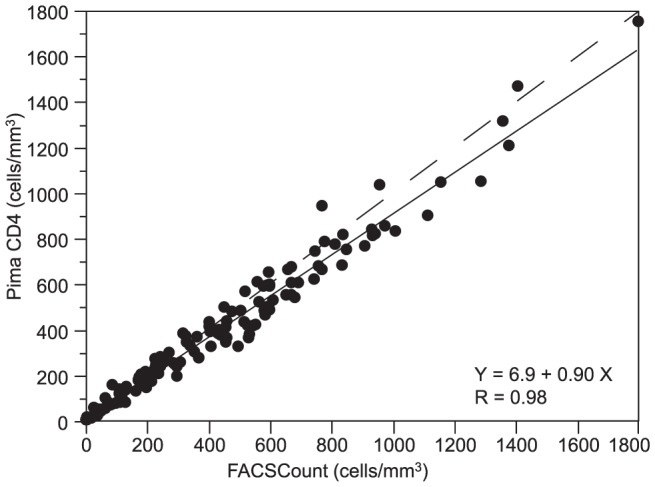
Passing-Bablok regression between Pima CD4 and FACSCount (N = 128). The x-axis represents CD4 counts provided by the FACSCount reference and the y-axis represents the CD4 counts provided by the Pima CD4. The solid line represents the regression line, and the dashed line represents the line y = x.

Passing-Bablok regression analysis showed an intercept of 15 cells/mm^3^ and a slope of 0.82 for CyFlow Counter (Figure 1), and an intercept of 7 cells/mm^3^ and a slope of 0.90 for Pima CD4 (Figure 2). The corresponding concordance correlation coefficients ρ_c_ were 0.94 for CyFlow Counter and 0.98 for Pima CD4. In HIV^+^ patients, we found good correlation and concordance between the alternative methods and the reference. CyFlow Counter showed better concordance and correlation with FACSCount in low and medium than in high CD4 (Table 2), while Pima CD4 showed similar results in the 3 different CD4 strata (Table 3).

**Table 2:** Comparison of CD4 T-cell counts between CyFlow Counter and FACSCount.

	All (N = 128)	HIV+ (N = 111)	Low CD4 (N = 39)	Medium CD4 (N = 38)	High CD4 (N = 51)
Intercept (95% CI) (cells/mm^3^)	15 (10 to 23)	15 (10 to 19)	10 (5 to 14)	−25 (−63 to 12)	48 (−5 to 91)
Slope (95% CI)	0.82 (0.81 to 0.84)	0.83 (0.81 to 0.85)	0.88 (0.81 to 0.96)	0.97 (0.86 to 1.09)	0.78 (0.71 to 0.85)
Concordance ρ_c_ (95% CI)	0.94 (0.92 to 0.95)	0.96 (0.94 to 0.97)	0.97 (0.94 to 0.98)	0.87 (0.77 to 0.92)	0.78 (0.69 to 0.85)
Pearson ρ	0.98	0.99	0.97	0.93	0.94
Accuracy C_b_	0.96	0.97	0.99	0.93	0.84
Bias (LOA) (cells/mm^3^)	−63 (−245 to 120)	−45 (−165 to 76)	0 (−30 to 30)	−33 (−97 to 31)	−133 (−349 to 84)
Relative bias (LOA)	−9.8% (−38.1 to 18.4)	−7.9% (−35.1 to 19.2)	2.2% (−28.1 to 32.5)	−10.6% (−29.0 to 7.8)	−17.5% (−39.2 to 4.2)
Mean Similarity (95% CI)	95.8% (94.5 to 97.1)	96.6% (95.3 to 98.0)	101.7% (98.9 to 104.6)	95.2% (93.8 to 96.6)	92.2% (90.8 to 93.6)
Relative SD	7.4%	7.3%	8.2%	4.5%	5.3%

95% CI means 95% of Confidence Interval; Low means CD4 lower than 200 cells/mm3; Medium means CD4 between 200 and 500 cells/mm3; High means CD4 greater than 500 cells/mm3; HIV+  =  HIV-infected patients, LOA  =  limits of agreement, SD  =  standard deviation.

**Table 3:** Comparison of CD4 T-cell counts between Pima CD4 and FACSCount.

	All	HIV+	Low CD4	Medium CD4	High CD4
Intercept (95% CI) (cells/mm^3^)	7 (4 to 20)	12 (5 to 25)	6 (−1 to 15)	4 (−79 to 64)	−85 (−153 to −17)
Slope (95% CI)	0.90 (0.87 to 0.94)	0.87 (0.84 to 0.92)	1.02 (0.86 to 1.13)	0.96 (0.76 to 1.19)	1.00 (0.91 to 1.09)
Concordance ρ_c_ (95% CI)	0.98 (0.97 to 0.98)	0.96 (0.95 to 0.97)	0.93 (0.87 to 0.96)	0.81 (0.67 to 0.90)	0.93 (0.89 to 0.96)
Pearson ρ	0.98	0.98	0.93	0.83	0.96
Accuracy C_b_	0.99	0.98	0.99	0.98	0.97
Bias (LOA) (cells/mm^3^)	−30 (−160 to 101)	−29 (−149 to 92)	6 (−39 to 51)	−18 (−117 to 81)	−65 (−224 to 93)
Relative bias (LOA)	−3.5% (−41.0 to 33.9)	−3.4% (−43.1 to 36.2)	7.3% (−44.4 to 59.0)	−5.2% (−34.4 to 23.9)	−9.7% (−32.7 to 13.3)
Mean Similarity (95% CI)	99.3% (97.3 to 101.3)	99.4% (97.2 to 101.7)	106.0% (100.2 to 111.8)	97.9% (95.7 to 100.2)	95.7% (94.1 to 97.2)
Relative SD	11.2%	11.9%	15.9%	7.1%	5.7%

95% CI means 95% of Confidence Interval; Low means CD4 lower than 200 cells/mm3; Medium means CD4 between 200 and 500 cells/mm3; High means CD4 greater than 500 cells/mm3; HIV+  =  HIV-infected patients, LOA  =  limits of agreement, SD  =  standard deviation.

**Figure 3:**
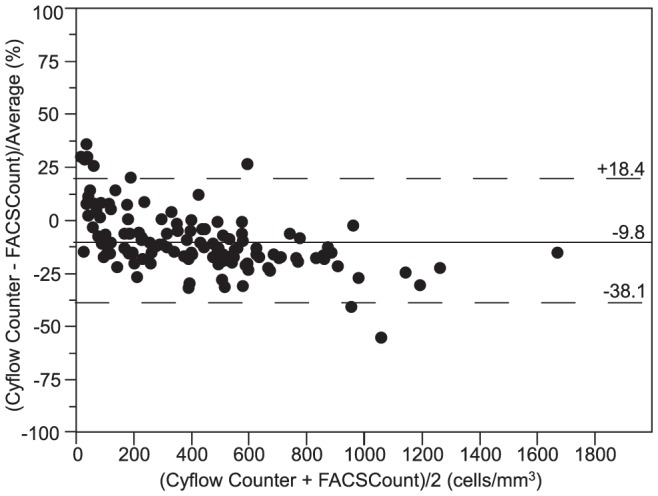
Pollock plot between CyFlow Counter and FACSCount (FACSCount >10 cells/mm^3^; N = 124). the x-axis represents the average of CD4 count from CyFlow Counter and FACSCount, and the y-axis represents the bias between CyFlow Counter and FACSCount divided by their mean. The solid line represents the mean bias, and the dashed lines represent the upper and lower limits of agreement (LOA  =  mean bias ±1.96 SD).

**Figure 4:**
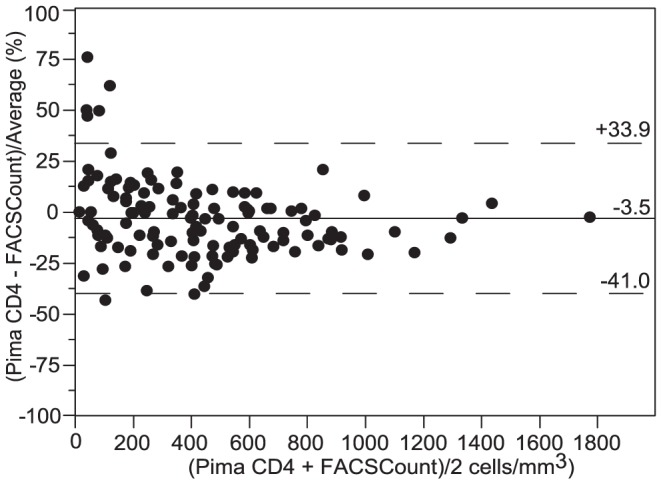
Pollock plot between Pima CD4 and FACSCount (FACSCount >10 cells/mm^3^; N = 124). the x-axis represents the average of CD4 count from Pima CD4 and FACSCount, and the y-axis represents the bias between Pima CD4 and FACSCount divided by their mean. The solid line represents the mean bias, and the dashed lines represent the upper and lower limits of agreement (LOA  =  mean bias ±1.96 SD).

By excluding samples with CD4 counts less than 10 cells/mm^3^ on FACSCount from data analysis (N = 4) to avoid interference of large relative differences which are not clinically relevant [17], we found a mean relative bias of −9.8% (−38.1 to 18.4) for CyFlow Counter (Figure 3), and −3.5% (−41.0 to 33.9) for Pima CD4 (Figure 4). Considering only HIV^+^ patients, CyFlow Counter showed a relative bias of −7.9% (−35.1 to 19.2), while Pima CD4 showed a relative bias of −3.4% (−43.1 to 36.2) similar to the relative bias of overall data. In low and medium CD4 strata, CyFlow Counter and Pima CD4 showed excellent agreement with FACSCount. In high CD4 stratum, both CyFlow Counter and Pima CD4 provided lower CD4 counts compared to those of FACSCount.

Considering only samples with CD4 T-cell counts more than 10 cells/mm^3^ on FACSCount (N = 124), the mean similarity (relative SD) was 96% (7%) and 99% (11%) respectively between CyFlow Counter and FACSCount, and between Pima CD4 and FACSCount.

Next, we determined the capacity of CyFlow Counter or Pima CD4 to correctly identify eligible patients for ART. At the WHO-2006 threshold of 200 cells/mm^3^, CyFlow Counter and Pima CD4 respectively showed a *kappa* coefficient (κ) of 0.903 and 0.902, with a sensibility of 97% (38/39) and 95% (37/39), and a specificity of 94% (68/72) and 96% (69/72). At the WHO-2010 threshold of 350 cells/mm^3^, CyFlow Counter and Pima CD4 respectively showed a coefficient κ of 0.909 and 0.873, with a sensibility of 100% (59/59) and 97% (57/59), and a specificity of 90% (47/52) and 90% (47/52). At the WHO-2013 threshold of 500 cells/mm^3^, CyFlow Counter and Pima CD4 respectively showed a κ of 0.730 and 0.758, with a sensibility of 100% (75/75) and 99% (74/75), and a specificity of 67% (24/36) and 72% (26/36).

### Comparison between CyFlow Counter and Pima CD4

Overall comparison between alternative methods showed good correlation (intercept of −6 cells/mm^3^, slope of 1.09) and excellent concordance (ρ_c_ of 0.95). Excellent correlation and concordance were shown on HIV^+^ patients (intercept  = 0 cells/mm^3^, slope  = 1.05, and ρ_c_ = 0.96). We found excellent correlation in low and medium CD4, and good concordance in low and high CD4 counts. Good agreement were shown between CyFlow Counter and Pima CD4 with a relative bias of 6.3% (−31.2 to 43.8) and similarity of 104% (10%). In HIV^+^ patients and in the different CD4 strata, the alternative methods showed excellent agreement between them (Table 4).

**Table 4:** Comparison of CD4 T-cell counts between Pima CD4 and CyFlow Counter.

	All	HIV+	Low CD4	Medium CD4	High CD4
Intercept (95% CI) (cells/mm^3^)	−6 (−19 to 2)	0 (−8 to 11)	−5 (−16 to 5)	13 (−47 to 78)	−151 (−286 to −59)
Slope (95% CI)	1.09 (1.05 to 1.14)	1.05 (1.00 to 1.09)	1.11 (0.94 to 1.30)	1.03 (0.82 to 1.21)	1.30 (1.15 to 1.55)
Concordance ρ_c_ (95% CI)	0.95 (0.94 to 0.96)	0.96 (0.94 to 0.97)	0.93 (0.87 to 0.96)	0.80 (0.65 to 0.89)	0.85 (0.77 to 0.90)
Pearson ρ	0.97	0.99	0.93	0.81	0.92
Accuracy C_b_	0.98	0.97	0.99	0.98	0.93
Bias (LOA) (cells/mm^3^)	33 (−132 to 203)	14 (−93 to 121)	5 (−46 to 57)	15 (−87 to 117)	67 (−169 to 303)
Relative bias (LOA)	6.3% (−31.2 to 43.8)	4.5% (−33.5 to 42.5)	5.2% (−46.0 to 56.5)	5.3% (−27.3 to 38.0)	7.8% (−21.7 to 37.3)
Mean Similarity (95% CI)	104.3% (102.4 to 106.2)	103.3% (101.3 to 105.4)	104.6% (99.4 to 109.8)	103.5% (100.6 to 106.3)	104.7% (102.3 to 107.1)
Relative SD	10.3%	10.5%	14.4%	8.5%	8.2%

95% CI means 95% of Confidence Interval; Low means CD4 lower than 200 cells/mm3; Medium means CD4 between 200 and 500 cells/mm3; High means CD4 greater than 500 cells/mm3; HIV+  =  HIV-infected patients, LOA  =  limits of agreement, SD  =  standard deviation.

**Figure 5:**
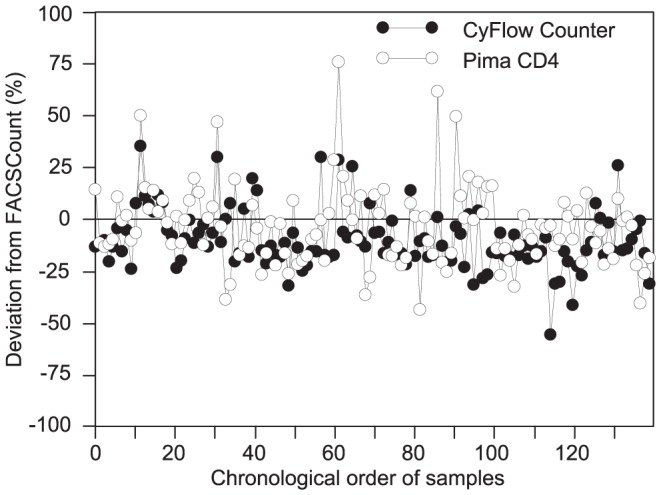
Bias comparison between CyFlow Counter and Pima CD4 from FACSCount results: Sample number in chronological order (FACSCount >10 cells/mm^3^; N = 124). the x-axis represents the chronological order of samples; the y-axis represents the relative bias of alternative methods from the FACSCount reference; the solid line represents the zero-bias.

**Figure 6:**
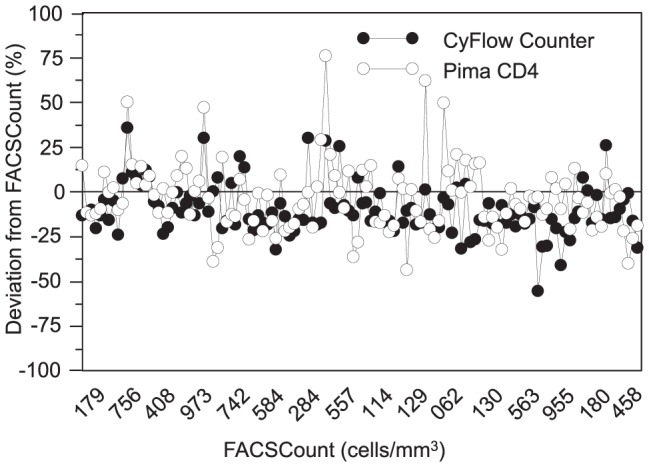
Bias comparison between CyFlow Counter and Pima CD4 from FACSCount results: samples with regard to their CD4 counts (FACSCount >10 cells/mm^3^; N = 124). the x-axis represents the CD4 counts from the FACSCount reference; the y-axis represents the relative bias of alternative methods from the FACSCount reference; the solid line represents the zero-bias.

We plotted both biases of alternative systems against the reference CD4 results. The deviation of alternative systems from FACSCount reference is not influenced by the chronological order of samples. Indeed, the biases observed the first days of the study were not different from those observed the last days due to a potential implementation period, so all samples were included (Figure 5). Either CyFlow Counter's deviation (r = −0.546, p<0.0001) or Pima CD4's deviation (r = −0.289, p = 0.0010) were inversely correlated with CD4 T-cell counts. In low CD4 counts, the alternative systems mostly provided higher CD4 counts than the FACSCount, CyFlow Counter's results were closer to FACSCount compared to Pima CD4. However, in medium and high CD4 counts, they mostly underestimated the CD4 counts compared to FACSCount. CD4 counts from Pima CD4 were closer to those of FACSCount compared to CyFlow Counter's CD4 counts which were the lowest. In some samples, CyFlow Counter and Pima CD4 were closer between them than compared to FACSCount (Figure 6).

## Discussion

Although several studies have evaluated the first version of the CyFlow Counter (CY-S-3021) using liquid reagents [29], [30], our study is the first to evaluate the latest version of CyFlow Counter (CY-S-3022) together with its new lyophilized CD4 reagents for absolute CD4 counts (CD4 Easy count kit-dry). Lyophilized reagents require less critical storage conditions and are thus more attractive for resources-limited countries. CD4 easy count kit-dry (Partec) and Pima cartridges (Alere) allow eliminating the need for cold chain.

Even if the FACSCount has filled a first gap towards affordable technology, cheaper instruments are required, and POC respond to this demand. To have an idea of the direct costs, in Senegal, the FACSCount instrument costs like €20,000 and reagents €6.91/test. CD4 counting by FACSCount requires additional consumables (control beads, sheath fluid, cleaning and rinsing solutions), cold chain for reagent shipment and storage, air conditioner, and maintenance service. CD4 counting using the CD4 easy count kit-dry is only €2.77/test, but the CyFlow Counter is expensive (€18,500), and requires additional consumables (sheath fluid, count check beads, cleaning and decontaminating solutions) and maintenance service. The Pima CD4 is more affordable (€9,200 the device), does not need additional consumables, and the training lasts only some hours. However, the cartridge cost is relatively high (€6.82/test).

Besides independent evaluation of those two instruments, our study also introduced an additional comparison approach. To date, a gold standard for CD4 T-cell enumeration does not exist, and evaluation of new emerging CD4 technologies is performed by comparison with the existing flow cytometry-based reference instruments. Since all technologies, including reference instruments, have limited precision and accuracy, discordant results are often difficult to attribute to one or other instrument. We compared the CyFlow Counter with the FACSCount, an established reference instrument for CD4 counting in the field, and with the Pima CD4, a new point-of-care CD4 instrument. The comparison of 3 CD4 technologies is highly interesting, as we noticed that in some samples, CyFlow Counter and Pima CD4 provided concordant results which were discordant on FACSCount.

The Pima CD4 can be used with capillary blood but for this comparison, we decided to use only venous blood so that the same blood sample could be analysed on the 3 different instruments. The use of capillary blood requires immediate availability of a Pima analyser, once blood is loaded on the Pima cartridge as it should be run within 5 minutes. In addition, our team and other investigators have shown better performance and lower rate of errors when the Pima CD4 system was operated with venous instead of capillary blood [28], [31], [32], so venous blood is recommended when possible. The FACSCount and the CyFlow Counter showed comparable mean intra-assay precision (CV<6%) which is in line with previous studies [8], [9], [17], [18], [29], [33]. However for lower CD4 counts, CyFlow Counter produced a less precise results (CV = 9.8%) than FACSCount (CV = 5.3%). The Pima CD4, based on fluorescence imaging optics, was found to be less precise than the flow cytometry based systems, in particular for CD4 counts below 200 cells/mm^3^ (CV = 17.6%) which is in line with published results [13]. However, the intra-assay variation of the Pima CD4 for low count, which was 29 cells/mm^3^, is within the acceptable standard deviation of 35 cells/mm^3^ determined by the company. For a count of 200 cells/mm^3^, 29 cells represents 14.5%. The precision of Pima CD4 was in line with the CV of 10.3% found in the study conducted in Kenya by Mwau and colleagues [34].

Despite its lower precision, Pima CD4 showed better correlation, concordance and similarity with the reference instrument (FACSCount) than the CyFlow Counter. Indeed, the distance of the regression line from the 45° line through the origin is larger for the CyFlow vs. FACSCount than for the Pima CD4 vs. FACSCount. CyFlow Counter and Pima CD4 showed acceptable agreement with the FACSCount. Surprisingly, and despite the higher precision of the CyFlow Counter, the absolute limits of agreement between CyFlow Counter and FACSCount were wider than between Pima CD4 and FACSCount. This is probably due to the systematic lower counts on CyFlow Counter than on FACSCount. Both alternative systems gave lower CD4 counts than FACSCount, but less pronounced on Pima CD4. Furthermore, the bias of CyFlow Counter appears to be affected by the higher CD4 counts and thus by the inclusion of samples from HIV-negative donors. On Pima CD4, HIV status did not have a negative impact on the accuracy and the concordance. The larger bias towards “underestimation” of CyFlow Counter may thus explain its higher sensibility (≥97%) to identify patients in need of ART as compared to Pima CD4 (≥95%) which is still very good. Applying the new 2013-WHO guidelines increased the sensibility (>99%) and lowered the specificity (67% for CyFlow and 72% for Pima). When applying the new 2013 guidelines together with the decentralization with the CD4 POC systems, so relying on these systems with lower specificity, even more patients will be treated than expected based on predictions from standard flow cytometers currently in use. Policy makers should take this fact into account to foresee extra ART stocks before recommending ART start at 500 cells/mm^3^. Previous studies that evaluated the first version of CyFlow Counter (CY-S-3021) or other CyFlow instruments (e.g. CyFlow Green, CyFlow Blue) showed similar results to this study [15], [17], [18], [29], [33], [35], [36], confirming the adequate performance of the newly available stabilized reagents on the most recent version of the CyFlow Counter (CY-S-3022). Only a few studies showed that CyFlow Counter and the CyFlow Green gave higher CD4 counts compared to the reference instruments [30], [37]. The study conducted in Uganda on CyFlow Counter showed too large limits of agreement to be clinically acceptable probably due to its large bias (+92 cells/mm^3^), and low sensibility (71%) due to the “overestimation” compared to FACSCan reference results [30], [37].

Our Pima CD4's results are in agreement with those reported by others comparing the Pima CD4 with FACSCalibur, FACSCan, FACSCount, Epics XL or CyFlow SL3 [14], [19], [31], [32], [38]–[40]. However, by duplicating measurements, other studies showed CVs ranging from 2% to 14% [13], [28]. Moreover, a recent study in Kenya showed lower performance than our [34]. The influence of different operators (2 to 9 sites) [31] and the use of capillary blood to determine the sensibility and specificity may explain the lower performance of Pima CD4 when compared to FACSCount or when compared to CyFlow SL3 [34].

Other alternative CD4 counting systems such as Guava EasyCD4, Apogee Auto40 and a microchip-based CD4 counting previously evaluated showed good precision (CV<6%) and good agreement compared to the reference systems [16], [20], [41], [42].

In summary, we demonstrated that either the CyFlow Counter or the Pima CD4 systems can accurately provide CD4 T-cell counts which showed an acceptable agreement with the results from the FACSCount. Thanks to their practical advantages, Partec CyFlow Counter and/or Alere Pima CD4 systems can be operated easily in resource-limited settings. Particularly, they can be used to screen HIV-patients to assess their eligibility for antiretroviral treatment (ART). Manufacturers could however still improve the performance on the CD4 POC systems especially for precision and in the reduction of the rate of errors. The performance of the use of capillary blood must be improved otherwise we highly recommend using venous blood. In any case, the POC systems must be less dependent on operators.

Regarding to daily capacity and costs (high investment but then low per sample), the CyFlow Counter operated with its lyophilized reagents could be suitable for rural laboratories with high HIV prevalence where they can handle large numbers of blood samples. The POC Pima CD4 system would be rather useful in rural laboratories with low sample throughput.
